# Elements of social accountability in undergraduate health sciences curricula: A scoping review

**DOI:** 10.4102/sajp.v82i1.2269

**Published:** 2026-02-09

**Authors:** Laeeqa Sujee, Vaneshveri Naidoo, Hellen Myezwa

**Affiliations:** 1 Department of Physiotherapy, Faculty of Health Sciences, University of the Witwatersrand, Johannesburg, South Africa; 2 School of Therapeutic Sciences, Faculty of Health Sciences, University of the Witwatersrand, Johannesburg, South Africa

**Keywords:** equity, community-oriented, curricula, relevance, cost-effectiveness

## Abstract

**Background:**

Social accountability represents the social contract between medicine and society, encouraging healthcare professionals (HCPs) to address social and health-related issues. The importance of integrating social accountability into curricula is widely recognised, but there is a lack of comprehensive mapping of the specific elements that should be included.

**Objectives:**

To identify the key elements of social accountability that should be integrated into undergraduate health sciences curricula to develop socially accountable HCPs.

**Method:**

The scoping review was conducted following the Joanna Briggs Institute Reviewers’ Manual 2015 for scoping reviews. A comprehensive search was employed using various keyword combinations and search strings, inclusive of published and grey literature from the past 15 years. Studies were systematically charted and analysed.

**Results:**

A rigorous screening process resulted in 47 studies being included in the review. Majority of the studies were qualitative, with the highest number of studies originating from Canada, South Africa, and the United States, as well as several multi-country studies. Equity emerged as the most frequently mentioned value, while cost-effectiveness was discussed the least.

**Conclusion:**

The scoping review demonstrates that embedding equity-driven approaches, community engagement, interprofessional collaboration and transformative learning in healthcare systems and tertiary institutions is vital. Addressing these priorities through undergraduate health sciences training can foster more inclusive, responsive and effective healthcare delivery, and improve health outcomes.

**Clinical implications:**

Integrating the identified elements of social accountability into undergraduate health sciences curricula may lead to improved patient outcomes, reduced health disparities, and more effective, patient-centred care.

## Introduction

Socially accountable healthcare professionals (HCPs) understand their responsibility to the community they serve through training in population health and addressing health inequities (Clithero-Eridon, Albright & Ross 2020). Social accountability is the social contract between medicine and society, indicating that HCPs must ensure their services are equitable and responsive to the person, the community and population needs (Ventres, Boelen & Haq 2018). Historically, HCPs have been required not only to heal individuals ethically but also to improve society’s health (Abdalla 2012). Recognising social accountability encourages continuous learning, professional development, community engagement, advocacy and cultural competence (Preston et al. 2016).

Effective practice requires transformative education curricula that prepare HCPs to meet country-specific needs through decolonial and equitable approaches (Boelen & Woollard 2009; Rispel 2023). Transformative education addresses global challenges such as discrimination, poverty, inequality, environmental degradation, loss of biodiversity and climate change (Green-Thompson, McInerney & Woollard 2017), with social accountability as a core principle (Romano 2017). Transformative education is imperative in developing countries to develop HCPs who can provide equitable, effective and accessible healthcare (Preston et al. 2016). Thus, tertiary institutions should ensure that curricula cultivate HCPs who maximise societal impact and resource use (Armstrong & Rispel 2015).

Social accountability in tertiary institutions means aligning education, research and services to improve the health status of the communities served (Abdalla et al. 2022). Such curricula promote teamwork, effective communication, HCP readiness, self-confidence, empowerment (Boelen & Woollard 2009) and community engagement (Mahdavynia et al. 2022). True social accountability transcends clinical competence to include equity, inclusion, non-discrimination and responsiveness to community needs (Clithero-Eridon et al. 2020).

The World Health Organization (WHO) defines social accountability through four core values: relevance, quality, cost-effectiveness and equity (Boelen & Heckman 1995), with equity serving as both a guiding value and a desired outcome. Equity ensures that healthcare services and educational curricula address disparities and promote fairness in access and outcomes by ensuring that all individuals, particularly those from underserved or marginalised populations, have access to cost-effective, quality and culturally sensitive services. For healthcare services, relevance means prioritisation of the most essential problems of the population; while for curricula, it involves aligning educational content with community needs (Boelen & Heckman 1995). Quality refers to the delivery of health services based on good practice and established standards, in a manner that is respectful, effective and efficient (Brown & Grierson 2022). The concept of efficiency is about using resources cost-effectively. Hence, social accountability cannot exist without actively reducing health inequities and preparing graduates to serve all communities justly.

To improve curricula in social accountability, it is essential to understand the key elements and integrate them. This scoping review maps evidence on the key elements of social accountability that should be included in undergraduate health sciences curricula to prepare graduates to meet the needs of their country. Searches of the *Cochrane Database of Systematic Reviews* and the *JBI Database of Systematic Reviews and Implementation Reports* found no scoping reviews or systematic reviews on this specific topic.

### Review question

What are the key elements of social accountability that should be included in undergraduate health science curricula to prepare graduates to meet the needs of their country?

## Research methods and design

The scoping review followed ‘the Joanna Briggs Institute Reviewers’ Manual 2015: Methodology for JBI Scoping Reviews’ (Peters et al. 2015). The scoping review was directed by the population, concept and context (PCC) approach (Peters et al. 2015). The population was not applicable. The concept focused on key social accountability elements to include in undergraduate health sciences curricula to prepare graduates for their country’s needs. The context was undergraduate health sciences education curricula globally.

### Inclusion criteria

The search included all published and grey literature describing social accountability elements for undergraduate health sciences curricula. It considered studies of all designs: quantitative, qualitative (narrative, phenomenological and grounded theory), mixed methods, observational studies (case-control studies, cohort studies, case series, cross-sectional studies, case reports), expert reviews and opinion pieces. No limitations were set on publication status.

### Exclusion criteria

Studies were excluded if not published in English or older than 15 years, to ensure applicability, relevance and currency in relation to evolving social accountability elements in undergraduate health sciences education. Studies involving postgraduate curricula or non-health science curricula (‘wrong population’) were excluded, as were studies lacking key elements of social accountability for curricula (‘wrong outcomes’).

### Search strategy

Table 1 outlines the characteristics of the database search for the scoping review.

**TABLE 1:** Search strategy.

Databases	Search string	Key words
**White literature:** Google Scholar, Semantic Scholar, ERIC, PubMed, PEDRO, Scopus, EBSCO Host (Cinahl and Medline) **Grey Literature:** Professional Association Websites, Department of Health websites, Databases for dissertations and theses, Open Grey and Grey Source Index	‘Social Accountability’ OR ‘Social Responsibility’ OR ‘Social Responsiveness’ AND ‘Health Sciences’ AND ‘curriculum’ OR ‘curricula’ OR ‘education’.	Public Health, population health, curricula, community, social accountability, social responsibility, equity, determinants of health, cost-effectiveness, ethics, relevance, social justice, transformation, community needs and quality.

### Study selection

A comprehensive search was conducted using various keyword combinations and search strings, filtered by year (last 15 years) and English language. The results are noted in Table 2. Records were screened by title, with relevant records imported into a reference manager, *Zotero*, as well as *Covidence*, which is web-based software designed to organise the screening, data extraction and quality assessment processes in systematic and scoping reviews. However, when titles lacked sufficient information, abstracts were reviewed to avoid excluding relevant articles. The reviewer focused on titles related to social accountability in health sciences using keywords from Table 1.

**TABLE 2:** Search strategy.

Search engine	Search string	Number of studies	Relevance	Identification process
ERIC	‘Social Accountability’ OR ‘Social Responsibility’ OR ‘Social Responsiveness’ AND ‘Health Sciences’ AND ‘curriculum’ OR ‘curricular’ OR ‘education’.	7	5 articles were relevant based on the title and were added to *Covidence* for review.	Studies were identified by title based on the inclusion criteria and looking for the inclusion of related keywords: Public Health Population health Curricula Community Social accountability Social responsibility Equity Determinants of health Cost-effectiveness Ethics Relevance Social justice Transformation Community needs Quality
	‘Social Accountability’ AND ‘Health Sciences’ AND ‘curriculum’.	23	4 articles were relevant based on the title and were added to *Covidence* for review.	
PEDRO	‘Social Accountability’ OR ‘Social Responsibility’ OR ‘Social Responsiveness’ AND ‘Health Sciences’ AND ‘curriculum’ OR ‘curricular’ OR ‘education’.	0 relevant	0 articles were relevant based on the search, thus none were added to *Covidence* for review.	
Scopus	‘Social Accountability’ OR ‘Social Responsibility’ OR ‘Social Responsiveness’ AND ‘Health Sciences’ AND ‘curriculum’ OR ‘curricular’ OR ‘education’.	44	30 articles were relevant based on the title and were added to *Covidence* for review.	
	‘Social Accountability’ AND ‘Health Sciences’ AND ‘curriculum’.	7	7 articles were relevant based on the title and were added to *Covidence* for review.	
PubMed	‘Social Accountability’ OR ‘Social Responsibility’ OR ‘Social Responsiveness’ AND ‘Health Sciences’ AND ‘curriculum’ OR ‘curricular’ OR ‘education’.	1 277 084	The search string was then refined and yielded results as noted in the line below.	
	‘Social Accountability’ AND ‘Health Sciences’ AND ‘curriculum’.	63	56 articles were relevant based on the title and were added to *Covidence* for review.	
Google Scholar	‘Social Accountability’ OR ‘Social Responsibility’ OR ‘Social Responsiveness’ AND ‘Health Sciences’ AND ‘curriculum’ OR ‘curricular’ OR ‘education’. ‘Social Accountability’ AND ‘Health Sciences’ AND ‘curriculum’.	Over 1 million	Google Scholar was not used as a database but Semantic Scholar was used instead to refine this search.	
Semantic Scholar	‘Social Accountability’ OR ‘Social Responsibility’ OR ‘Social Responsiveness’ AND ‘Health Sciences’ AND ‘curriculum’ OR ‘curricular’ OR ‘education’.	493	81 articles were relevant based on title and were added to *Covidence* for review.	
	‘Social Accountability’ AND ‘Health Sciences’ AND ‘curriculum’.	4500	The search string above was used as it yielded more relevant studies.	
Open Grey	Combinations of search strings above.	0 relevant	0 articles were relevant based on the search, thus none added to *Covidence* for review.	

Duplicates were removed using *Covidence* software and manual checking. The first and second authors independently screened abstracts against inclusion criteria and Table 1 keywords to identify relevant studies, excluding those that did not qualify. The third author was the third reviewer and resolved conflicts which were based on disagreements regarding the appropriateness of the studies to be included, or if the first two reviewers were undecided, as *Covidence* allows a ‘yes’, ‘no’ and ‘maybe’ option when reviewing. Subsequently, the first author conducted a full-text screening of all articles. The selection process, including the numbers of included and excluded studies, is illustrated in a Preferred Reporting Items for Systematic Reviews and Meta-analysis (PRISMA) flow chart (Ramasamy 2022).

### Data extraction

In scoping reviews, data extraction is referred to as ‘charting’, where a descriptive summary of the results is developed (Ghalibaf et al. 2017). Microsoft Excel charting forms were designed through team consultation to collect relevant data (Pollock et al. 2023). Extracted information included title, authors, publication date, study location and country in which the study was conducted, study setting (e.g. community or university), study design and population, and social accountability elements addressed. The first author completed charting through an iterative process with input from the co-authors (Pollock et al. 2023). The data are presented descriptively using figures and tables and synthesised narratively.

### Ethical considerations

Ethical clearance was obtained from the University of the Witwatersrand Human Research Ethics Committee (Medical) (ethics clearance number: M240905).

## Results

### Study inclusion

**FIGURE 1:**
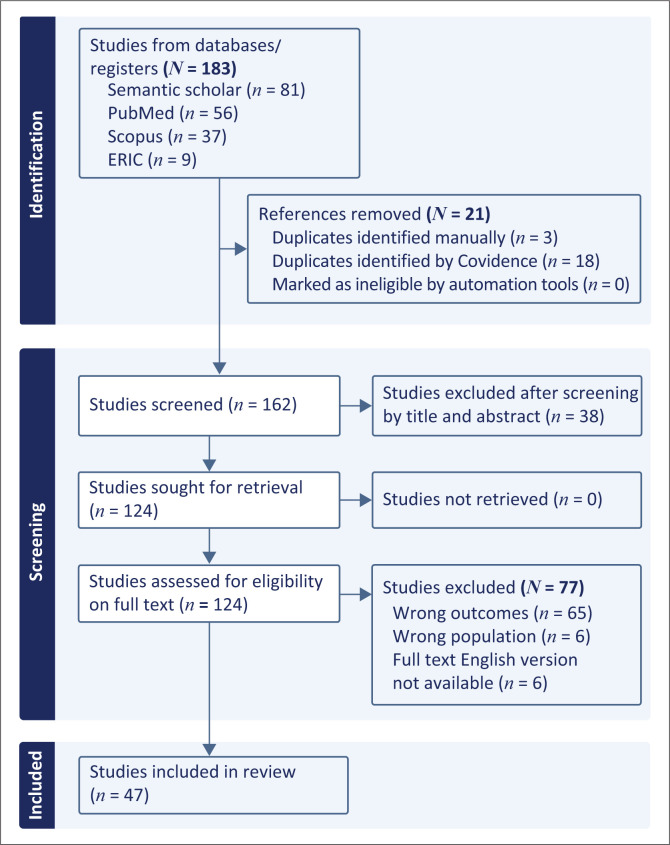
Preferred Reporting Items for Systematic Reviews and Meta-analysis chart for scoping review.

The identification phase began with 183 studies selected by title screening based on inclusion criteria and keywords from Table 1, and were then imported into *Covidence*. After removing 21 duplicates, 162 studies underwent abstract screening by two reviewers, with a third reviewer resolving conflicts based on the appropriateness of the studies. A total of 124 studies were screened by full text, resulting in 47 studies included in the final review. The screening process by full text identified whether the study recommended various elements of social accountability that should be included in undergraduate health sciences curricula or not. The 47 included studies specifically recommended key elements of social accountability to be incorporated into undergraduate health sciences curricula. Figure 1 outlines this information through a PRISMA chart.

### Characteristics of the studies

Information from the studies was extracted and populated into a charting form (Table 3). The countries or regions where the studies were conducted are grouped according to the World Bank income levels. Nineteen studies were from high-income countries, 11 from upper-middle-income, 5 from low-middle-income and 2 from low-income countries. Four studies were conducted across more than one country (studies involved multiple specific countries, often within a region with defined contexts) and another four were conducted globally (took a global perspective and was not limited to specific countries). Additionally, one study focused on Latin America and one on the Eastern Mediterranean region (exact countries included in these regions were not specified).

**TABLE 3:** Study characteristics.

Article number	Title	Reference	Country/Region	Study design	Population and/or sample	Setting	Elements of social accountability for curriculum development
1	Social accountability: A survey of perceptions and evidence of its expression at a sub-Saharan African university.	Galukande, Nakasujja and Sewankambo (2012)	Uganda	Cross-sectional descriptive qualitative study.	12 participants who were either senior medical educators or students.	One health sciences college in Uganda.	Efficient use of resources and community development.
2	Introducing first year students to inter-professionalism: Exploring professional identity in the ‘enterprise culture’: A foucauldian analysis.	DeMatteo and Reeves (2013)	Canada	Qualitative via three open ended questions and then focus groups: A foucauldian analysis.	234 students were interviewed. Focus Groups included 30 students.	Canadian university.	Professional identity, interdisciplinary teamwork, removal of hierarchies, efficiency, patient empowerment, importance of improving healthcare system by ‘responsibilising oneself’, improving patient care through understanding of other professions, awareness raising of one’s own profession, patient-centred care, improved communication with patients/families and other healthcare professionals.
3	Social accountability of medical schools and academic primary care training in Latin America: Principles but not practice.	Puschel et al. (2014)	Global	Mixed methods approach that combined a qualitative thematic analysis with a quantitative ecological design.	26 studies were selected for the thematic literature review. Medical schools from nine Latin American and non-Latin American countries were included in the quantitative ecological design.	9 Latin American and non-Latin American medical schools.	Networking, community-oriented programmes, ethical responsibility, emphasis on primary healthcare, distributive justice.
4	Gaps in studies of global health education: An empirical literature review.	Liu et al. (2015)	Global	Empirical literature review.	238 studies were included.	Global health education in medical curricula.	Global Health, understandings around ‘health for all’ and ‘health equity’, interdisciplinary collaboration, the definition and scope of global health, the demands on global health education of medicine-related students in developing countries, the challenges and opportunities associated with interinstitutional or interprofessional collaborations and the evaluation of global health education.
5	Social accountability and nursing education in South Africa.	Armstrong and Rispel (2015)	South Africa	Qualitative via in-depth interviews.	44 key informants were selected.	South African healthcare sector.	The link between professional competencies and patient and population health priorities, teamwork, emphasis on primary health care, leadership, understanding of the health system, community participation and community-based, interprofessional education based on principles of primary healthcare and focusing on the social determinants of health, social and communication skills, application of theoretical knowledge to patient care.
6	Developing social accountability in 1st-year medical students: A case study from the Nelson R Mandela school of medicine, Durban, South Africa.	Van Wyk et al. (2016)	South Africa	Case study: Qualitative document analysis.	249 first-year medical students.	Medical curriculum at one university in KwaZulu-Natal, South Africa.	Early exposure to experiential learning, social determinants of health, interpersonal skills, empathy and compassion.
7	An investigation on social accountability of general medicine curriculum.	Emadzadeh et al. (2016)	Iran	Cross-sectional study completed in three phases.	19 participants completed all three phases.	Iranian health centres (public health sector).	Epidemiology and disease trends, disease burden in the community, clinical skills, communication and leadership skills, principles of prevention, screening, diagnosis, treatment and rehabilitation of patients in the community, rural health, experiential learning, referral systems, advocacy, community oriented medical education, social determinant of health familiarising students with insurance systems and people working in charities, primary healthcare, professionalism, accountability, altruism, and empathy.
8	Toward diversity-responsive medical education: Taking an intersectionality-based approach to a curriculum evaluation.	Muntinga et al. (2016)	The Netherlands	Mixed-methods study.	Eight stakeholders were interviewed.	Medical curriculum in the Netherlands.	Socio-cultural and biomedical aspects of diversity. Patient safety, communication, ethics and law, and inter-culturalisation and diversity, social justice, reflexivity, social determinants of health.
9	Improving community health using an outcome-oriented CQI approach to community-engaged health professions education.	Clithero et al. (2017)	United States, Australia, South Africa	Descriptive programme implementation study, or model-based case study.	N/A	Health workforce education institutions.	Professional identity, leadership skills, needs of vulnerable populations, prioritisation of needs, principles of primary healthcare.
10	The elephant in the room: Talking race in medical education.	Sharma and Kuper (2017)	Canada	Opinion piece.	N/A	Canadian medical education.	Social determinants of health, moving beyond race as a biological characteristic and understanding its complex social aspects. Race consciousness and reflexivity.
11	Doctors without borders.	Wass and Southgate (2017)	Global	Opinion piece.	N/A	Global health.	Global health as part of a philosophy of population needs, human rights, equity, and justice. Patient-centred communication. Resilience, leadership, flexibility, and the ability to cope with uncertainty are needed to tackle the complexities of current, as well as future, health care.
12	The social accountability of doctors: A relationship-based framework for understanding emergent community concepts of caring.	Green-Thompson et al. (2017)	South Africa	Qualitative study via focus groups.	81 community members were included in focus groups.	Communities in the North West, Mpumalanga and Gauteng provinces of South Africa.	Compassion and relationship building, community orientation and understanding specific needs, social determinants of health, Batho Pele Principles, ubuntu as engagement of the community, building therapeutic relationships.
13	The impact of socially-accountable, community-engaged medical education on graduates in the Central Philippines: Implications for the global rural medical workforce.	Siega-Sur et al. (2017)	The Philippines	Quantitative study via surveys.	152 graduates.	Two medical schools in the Philippines.	Community based curriculum including emersion into the community, rural health education, stakeholder engagement.
14	The impact of socially-accountable health professional education: A systematic review of the literature.	Reeve et al. (2017)	The Philippines/Australia	Systematic review.	22 studies included.	Studies globally which focus on social accountability in medical education.	Community engagement, experiential learning, rural development, equity and diversity, inclusion of longitudinal learning environments.
15	Social accountability: A framework for medical schools to improve the health of the populations they serve.	Rourke (2018)	Canada	Commentary piece.	N/A.	Medical education in Europe.	Curriculum must be relevant to the unique geographic, social, and cultural context and the priority health needs of the school’s community, region, and nation. Diversity and community-based learning, emphasis on underprivileged and underserved populations, Primary healthcare.
16	Social determinants of health: A pedagogical framework for advancing the Citizen Scholar.	Velardo (2018)	Australia	Descriptive study: Case study.	147 first-year undergraduate students enrolled in the ‘Social Determinants of Health’ course.	Public Australian university: First-year undergraduate course.	Social determinants of health, inequities, health literacy, citizen scholar- creativity, resilience, teamwork, practical advocacy to invoke action, empathy, fairness and social justice, politics of health, health advocacy, education around marginalised populations, experiential learning and action-oriented curricula.
17	Envisioning a socially accountable doctor: A three-axis curriculum emerging from final-year medical student reflections.	Green-Thompson, McInerney and Woollard (2018)	South Africa	Qualitative study.	25 medical students.	South African medical school.	Community orientation, primary healthcare, advocacy, compassion, equity and learning to share responsibility.
18	Time for action: Key considerations for implementing social accountability in the education of health professionals.	Ventres et al. (2018)	United States	Reflective article.	N/A.	Undergraduate health sciences schools.	Cultural transformations within health professional education ensuring a commitment to health equity and incorporating values as generosity, solidarity, and social interdependency when teaching how social determinants affect health, moving beyond intention to action-based strategies, primary healthcare, community-based interventions. Four key points: Partnerships, accreditation, competencies and leadership.
19	Transformation of medical education through Decentralised Training Platforms: A scoping review.	Mlambo et al. (2018)	Global	Scoping review.	59 studies were included.	Decentralised Training Platform (DTP) strategies for medical education internationally.	Rural health, community-based education, distributed community-engaged learning, primary healthcare and community-based services, medical partnerships and collaborations, home visits, professionalism and communication skills.
20	Toward interprofessional service-learning and social accountability in health: One South African University’s process-oriented-participatory journey.	Du Toit et al. (2019)	South Africa	Descriptive study: Case study.	N/A.	Health sciences university in South Africa.	Community engagement, experiential learning, social constructivism, social network development, professionalism, decolonisation of healthcare, intra- and inter-personal skills, leadership skills, advocacy, cost-effectiveness.
21	Social Accountability of a medical college in Pakistan – A case study.	Rahman, Khan and Mashaddi (2019)	Pakistan	Qualitative study via interviews.	21 faculty members were interviewed.	One medical college in Pakistan.	Prioritisation of community health outcomes, primary healthcare, focus on health promotion and prevention, cost-effective use of resources, transformative education.
22	Accountability in medical education from theory to practice Tabriz 2018 statement: A step towards the implementation of this social necessity.	Ghaffari et al. (2020)	Iran	Conference statement.	N/A.	2018 conference statement related to social accountability at a medical school in Iran.	Community engagement, interdisciplinary work, stakeholder collaboration, selective use of resources, basic principles of quality, equity, communication, and effectiveness, active participation in the development of the health system, social determinants of health, levels of healthcare, in-service training and continued development of HCPs, empathy and emotional aspects of the profession, person-centred care.
23	The DISCuSS model: Creating connections between community and curriculum – A new lens for curricular development in support of social accountability.	Goez et al. (2020)	Canada	Longitudinal quantitative curriculum review.	Student feedback surveys.	One medical university in Canada.	Community engagement, sexual and gender-oriented health, global health, alternative healthcare, human trafficking, indigenous health, refugee health, addiction medicine, diversity and health disparity topics.
24	Social accountability and nursing education in Kerala.	Sudha, Thomas and Jose (2020)	India	Qualitative (interviews and focus groups).	60 participants were included.	Kerala State.	Primary prevention, capacity building, community orientation, transformative teaching strategies, collaboration and partnership, capacity building.
25	Attitudes of medical students towards men who have sex with men living with HIV: Implications for social accountability.	Dunbar et al. (2020)	Haiti	Qualitative via in-depth interviews.	22 medical students.	Medical School in Haiti.	Sexual health and specificities of sexual minorities, inclusivity, equity and quality of medical services, breaking stigma, non-discrimination, moral responsibility, equity.
26	Addressing the health advocate role in medical education.	Boroumand et al. (2020)	Canada	Report.	N/A.	One university in Canada.	Health advocacy, social determinants of health, cultural competence, practical community-based learning opportunities, experiential learning, increasing connections between community organisations, healthcare providers, and healthcare trainees.
27	Social accountability across cultures, does the concept translate? An explorative discussion with primary care colleagues in Japan.	Ramsay, Stanyon and Takahashi (2020)	Japan	Review/opinion piece.	N/A.	Japanese healthcare sector.	Understanding society’s needs, partnering with stakeholders and those in public office, continuous education and learning, community-based practice, emphasis on primary health care, efficiency of services and resource use.
28	Telemedicine in long-term elderly care facilities as ‘social accountability’ in the context of COVID-19.	Bertasso et al. (2021)	Brazil	Experience report.	N/A.	Frail care facilities in Brazil.	Anticipation of society’s health needs, partnerships and collaboration, adapting to evolving roles, fostering outcome-based education, creating responsive and responsible governance of the medical school, refining the scope of standards for education, research and service delivery, supporting continuous quality improvement in education, research and service delivery, preventative care and early identification, efficient use of resources.
29	Teaching about racism in medical education: A mixed-method analysis of a train-the-trainer faculty development workshop.	Edgoose et al. (2021)	United States	Mixed methods study.	49 participants were included and were made up of those who teach Family Medicine.	Train-the-trainer workshop at the 2017 Society of Teachers of Family Medicine Annual Spring Conference, United States.	Equity, disparity statistics, cultural competence, and social determinants of health. Ensuring race is not taught as a biological construct, addressing systems and behaviours that result in resource limitations opposed to just a lack of resources. Racism, privilege and implicit bias.
30	Perceptions of faculty toward ‘social obligation’ at an Indian medical school.	Dandekar, Mhatre and Mohanna (2021)	India	Qualitative via interviews.	17 faculty members.	One medical school in India.	Social and cultural awareness of communities, partnerships and collaborations within the community, empathy, communication skills, ethics, professionalism, resource management.
31	Twelve tips to centre social accountability in undergraduate medical education.	Fung and Ying (2021)	Canada	Review.	N/A.	Canadian medical schools.	Community engagement and partnerships, international collaborations and partnerships, social determinants of health, experiential learning, personal reflection and discussions with diverse populations, interdisciplinary teamwork, diversity and inclusivity, rural health and primary health care.
32	Social accountability in undergraduate medical education: A narrative review.	Mihan et al. (2022)	Canada, Australia, New Zealand, United States, and the United Kingdom	Narrative review.	40 studies for descriptive analysis.	Undergraduate medical education.	Service learning and social determinants of health, hands-on experience among marginalised populations, community engagement and advocacy, mentoring in disadvantaged populations, rural health.
33	Reimagining clinical psychology.	Goghari (2022)	Canada	Opinion piece.	N/A.	Canadian Psychology schools.	Importance of diversity, cultural differences, cultural humility and reflexivity, encourage life-long learning and development.
34	Mapping health, social and health system issues and applying a social accountability inventory to a problem-based learning medical curriculum.	Kelly, Hyde and Abdalla (2022)	Ireland	Qualitative content analysis.	45 documents related to a medical school’s PBL curriculum were identified and analysed.	Irish medical school.	Traveller health, LGBTQI health, alcohol use, climate change, health and social issues, patient-centredness, cost-effectiveness, shared decision-making, professionalism and multidisciplinary healthcare teams, relevant health concerns, social determinants of health, health promotion and preventative measures, psychosocial issues in healthcare, health management issues, medical professionalism, referrals, multidisciplinary approaches, environmental impact on health, stakeholder management, understanding of the health system, continued learning of HCPs. Socio-economic circumstances of patient and community, person-centred care, treatment costs and cost-effective treatment plans, stakeholder management, cultural issues such as language and religion.
35	The training of a new socially responsible generation of health professionals with a patient-centred vision.	Lopez et al. (2022)	Latin America	Review.	N/A.	Latin America.	Telehealth technologies, efficient resource management to reach remote areas and disadvantaged populations, human dignity and person-centred care, ethics and professionalism, determinants of health, primary health care, orientation to the care of patients, orientation to the care of the work environment, and adaptation to the norms of the profession.
36	What makes a medical school socially accountable? A qualitative thematic review of the evaluation of social accountability of medical schools in the Eastern Mediterranean Region.	Abdalla et al. (2022)	Eastern Mediterranean region	Thematic review.	Three studies were thematically reviewed.	Eastern Mediterranean region.	Stakeholder communication, community-oriented education, primary healthcare, regional health issues, social determinants of health issues and cost–effectiveness in both education and research, quality of services.
37	Exploring the social accountability challenges of nursing education system in Iran.	Ezzati et al. (2023)	Iran	An exploratory descriptive qualitative design.	21 participants from the nursing society.	Hospitals, nursing schools, centres, universities and faculties.	Community-based curriculum with focus on professional skills, communication skills, self-confidence, knowledge and awareness, critical thinking skills, and teamwork skills, practical learning within the curriculum, target-oriented and client-centred education, early interaction with the community, efficient use of human and physical resources, health prevention.
38	Structural competence and equity-minded interprofessional education: A common reading approach to learning.	Bates et al. (2023)	United States	Mixed-methods evaluation.	223 students across multiple health professions attending three different universities in part one and 56 students in the follow-up stage.	Three universities in the United States.	Interprofessional education, social determinants of health, structural competency, values and ethics, teamwork, interprofessional communication, responsibility, awareness of unconscious biases, diversity and inclusivity.
39	Rejecting a narrative of individual deficit: a model for developing antiracist curriculum in the health sciences.	Samarron Longorio et al. (2023)	United States	Opinion piece.	N/A.	United States health sciences education.	Social and structural determinants of health, advocacy, social/cultural/critical theories, experiential knowledge, learning about marginalised populations, race and racism, health disparities, reflective practice.
40	Pathways, journeys and experiences: Integrating curricular activities related to social accountability within an undergraduate medical curriculum.	Dubé et al. (2024)	Canada	Qualitative descriptive study via focus groups.	62 participants included in focus groups.	Canadian university and community around it.	Social justice, experiential learning, advocacy, awareness of lived realities, understanding of communities and available resources, empathy and compassion, patient and community advocacy, stakeholder engagement and community collaboration.
41	Informing critical indigenous health education through critical reflection: A qualitative consensus study.	Rame et al. (2023)	Canada	Qualitative study design.	23 participants including students, staff and faculty working across a Canadian medical school.	Canadian Medical Schools.	Anti-racism, understanding of indigenous cultures and people, equity, indigenous health education, oppression and discrimination, social justice, structural and social determinants of health, decoloniality, health systems and policy, Impact and intentionality.
42	Understandings and practices: Towards socially responsive curricula for the health professions.	Hansen et al. (2023)	South Africa	Qualitative study design using focus groups and in-depth interviews.	101 participants who were health professional educators (24 focus groups and 47 in-depth interviews).	Six institutions in South Africa that offer undergraduate health sciences degrees.	Critical consciousness, health inequities, social determinants of health, communication skills, practical and experiential learning, clinical competencies, empathy, importance of self-reflection, understanding the health system, primary healthcare, interdisciplinary teamwork, biopsychosocial approach, self-awareness.
43	Teaching compassion for social accountability: A parallaxic investigation.	Cheu et al. (2023)	Australia, Canada, United States	Qualitative via in-depth interviews and workshops.	25 participants who were students and staff at a medical school.	Four medical schools across Australia, Canada, United States.	Being a health advocate, servicing underserved populations, community participation and involvement, stakeholder collaboration, bottom-up approach, addressing specific health needs, equity, social justice, inclusivity, care of populations instead of individuals alone, effective communication and therapeutic relationships.
44	Exploring the development of a framework of social accountability standards for healthcare service delivery: A qualitative, multipart, multimethods process.	Anawati, Cameron and Harvey (2023)	Canada	Qualitative, multipart, multimethods study.	7 participants from diverse backgrounds.	Canadian medical school and a tertiary, regional academic health sciences centre.	Professional skills, communication skills, self-confidence, knowledge and awareness, critical thinking skills, teamwork skills, practical learning within the curriculum, target-oriented and client-centred education, early interaction with the community, efficient use of human and physical resources, health prevention.
45	Assessing social accountability perspectives among Syrian medical students: A cross-sectional study.	Swed et al. (2023)	Syria	Quantitative study via surveys.	1312 medical students.	Syrian medical students.	Community-oriented partnerships, collaborative training initiatives addressing health disparities, stakeholder involvement, diversity and understanding the population.
46	Recognising stereotypes and their impact on health: A transformative learning activity for undergraduate health science students.	Hall (2024)	United States	Case study.	N/A.	Undergraduate health sciences schools.	Transformative learning, awareness of implicit biases/stereotypes and confrontation of these, critical self-reflection, cultural awareness and diversity, compassion, critical thinking, empathy, community leadership, social justice, inclusivity.
47	Community service rehabilitation therapist’s understanding of social accountability.	Msomi and Ross (2024)	South Africa	Qualitative study via interviews and focus groups.	27 community service rehabilitation therapists.	KwaZulu-Natal province in South Africa.	Primary healthcare, community-based rehabilitation, efficient use of resources, transparency within practice, community-based education, community involvement, communication skills including empathy and compassion, resourcefulness, collaboration, community assessment.

Note: Please see full reference list of this article, Sujee, L., Naidoo, V. & Myezwa, H., 2026, ‘Elements of social accountability in undergraduate health sciences curricula: A scoping review’, *South African Journal of Physiotherapy* 82(1), a2269. https://doi.org/10.4102/sajp.v82i1.2269, for more information.

HCPs, healthcare professionals; N/A, Not applicable, PBL, Problem-based learning, LGBTQI, Lesbian, Gay, Bisexual, Transgender, Queer/Questioning, Intersex.

**FIGURE 2:**
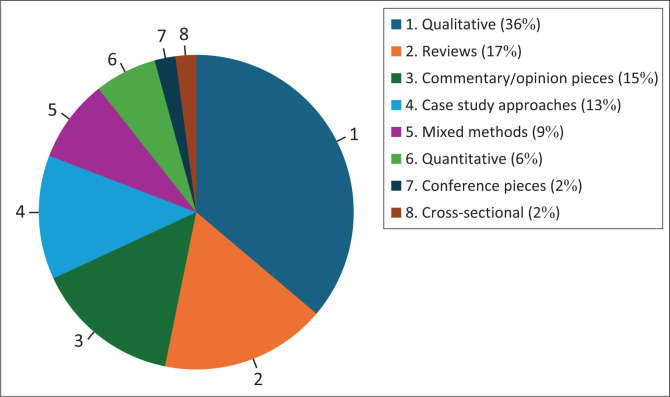
Study designs.

The review includes studies of various study designs. The majority of the studies were qualitative studies (*n* = 17), followed by reviews (*n* = 8), commentary and/or opinion pieces (*n* = 7), case study approaches (*n* = 6), mixed methods (*n* = 4), quantitative studies (*n* = 3), conference pieces (*n* = 1) and cross-sectional studies (*n* = 1). These are presented in Figure 2 as percentages.

### Review of findings: Elements of social accountability

**FIGURE 3:**
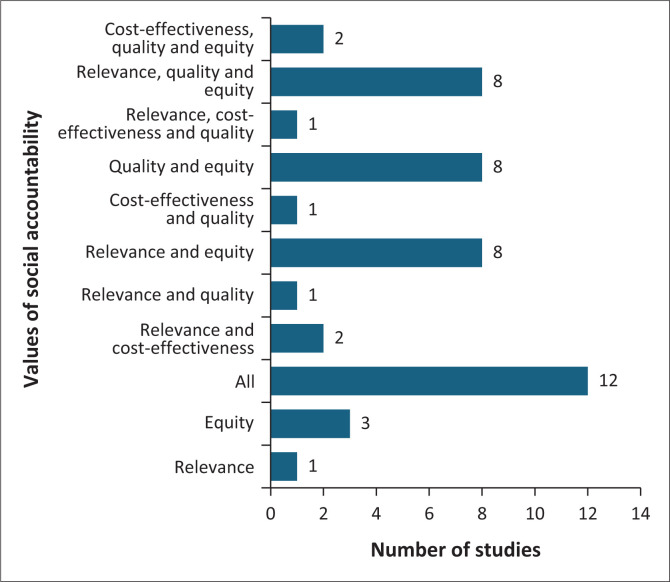
Values of social accountability noted in studies (*N* = 47).

A total of 47 studies were included in the review, highlighting key elements of social accountability for undergraduate health sciences curricula. Figure 3 illustrates the number of studies addressing each element or combination of elements.

**FIGURE 4:**
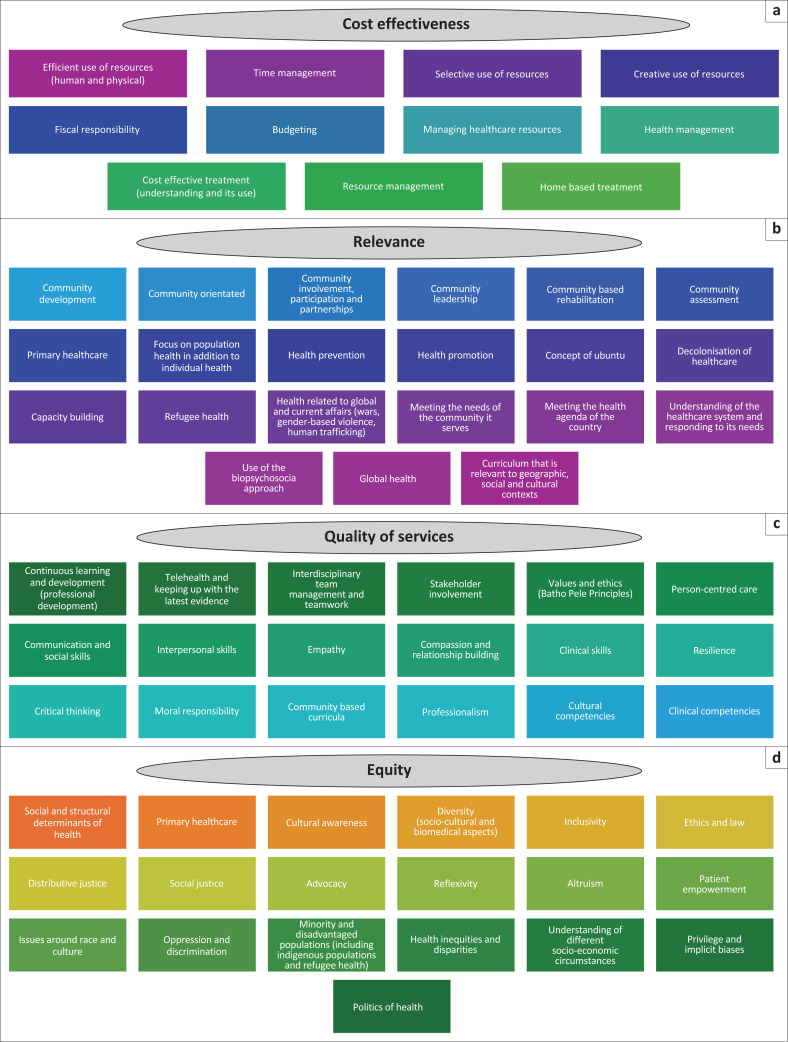
Elements of social accountability within each of the four core values: (a) cost effectiveness, (b) relevance, (c) quality of services and (d) equity.

The scoping review identified that 26% of the studies reviewed (12 out of 47) addressed all values of social accountability. Other frequently mentioned values included the combination of relevance and equity, the combination of quality and equity, and the combination of relevance, quality and equity (each with eight studies), while fewer studies focused on individual values such as cost-effectiveness or equity alone. Figure 4 describes elements that should be covered within each value of social accountability, namely, cost-effectiveness, relevance, quality and equity.

Under the value of cost-effectiveness, the studies identified elements such as the efficient use of physical, financial and human resources, budgeting, responsible management, creativity and accountability. Interventions promoting cost-effectiveness and sustainability involved group-based rehabilitation, home-based rehabilitation and caregiver training.

The studies identified the value of relevance to include elements that ensure HCPs deliver services aligned with the needs of the communities that they serve. Key aspects included in the studies were community participation, community inclusivity, and active involvement of community members in programme planning and implementation. In addition, understanding national health needs and priorities, incorporating health promotion and disease prevention, decolonising healthcare, and addressing the needs of marginalised and underserved populations were emphasised.

Quality was associated with curricula that foster both clinical and cultural competence. The studies demonstrated that emphasis must be placed on continuous professional development, evidence-based practice and person-centred care. Person-centred care was linked to attributes such as professionalism, communication and interpersonal skills, empathy, and compassion. Additional elements included collaborative practices, stakeholder engagement and policy involvement aimed at building resilient health systems.

The studies identified the need for HCPs to engage with the social and structural determinants of health affecting both the individuals and the communities that they serve. Identified elements within the value of equity include diversity, inclusivity, advocacy and social justice. Equity was presented in the studies concerning a broader understanding of the political, socio-economic, racial and cultural realities of the communities that HCPs serve.

Several cross-cutting elements, not exclusive to one value, also emerged as relevant to fostering social accountability among HCPs. These included interdisciplinary teamwork, collaboration, networking, stakeholder involvement, including global partnerships and leadership skills. Curriculum elements such as the dismantling of hierarchies, transformative learning and decoloniality were also highlighted. Additional themes such as awareness of implicit biases, stereotypes, self-reflection, transparency, experiential learning, environmental accountability and professional identity formation were all emphasised to develop socially accountable HCPs.

Community orientation featured prominently across the reviewed studies. Community-based learning including community assessment and participation, as well as aligning clinical practice with community health needs, was recommended. The results of the studies indicated the importance of embedding partnerships, community empowerment and community-based collaborations within the curriculum.

Global health literacy, inclusive of knowledge related to population health, health policies and national targets, was also identified as a necessary component within undergraduate health sciences curricula. Some studies noted that collaboration and partnerships should extend to international partners, such that HCPs improve their networking skills. The development of skills related to one’s specific profession, communication skills, social and interpersonal skills, as well as the ability to work within an interdisciplinary team, were all emphasised. Finally, the results of the studies indicated that quality extends beyond clinical competence to include awareness of environmental circumstances. This included teaching on social contexts, socio-economic conditions, race, racism, diversity, ethics and advocacy.

The review of literature identified transformative learning, decoloniality, leadership development and self-reflection as important elements to develop socially accountable HCPs. Experiential learning with active work in the community, as well as outreach activities, was emphasised to bridge the gap between theory and practice. Overall, the findings demonstrate that each of the four values of social accountability, namely, cost-effectiveness, relevance, quality and equity, encompasses multiple elements as presented in Figure 4.

## Discussion

The scoping review identifies a comprehensive range of themes as outlined in Figure 4 for integrating social accountability into undergraduate health sciences curricula. The findings underscore that embedding social accountability enhances the commitment of HCPs to population health. Integrating community-oriented, equity-driven, and interdisciplinary approaches into education, practice, and policy ensures that HCPs are equipped to address the needs of marginalised populations as well as advocate for social conditions that impact health outcomes. The review also demonstrated that a socially accountable HCP collaborates with partners and policymakers to create a truly accountable and resilient healthcare system.

### Professional development and competency frameworks

Ensuring high-quality health services requires a strong foundation in clinical competencies and evidence-based medicine. Accreditation bodies such as the Health Care Professional Council of South Africa (HPCSA) emphasise professionalism, good communication skills, lifelong learning, advocacy, scholarship and teamwork (HPCSA 2023). Frameworks such as the Canadian Medical Education Directives for Specialists Framework (CanMEDS) provide structured guidance on competencies for graduates, grouping abilities into roles; namely, medical expert, professional, communicator, collaborator, leader, health advocate and scholar (eds. Frank, Snell & Sherbino 2015; Royal College of Physicians and Surgeons, Canada 2024). Healthcare Professionals must be clinically competent and socially accountable to address complex and evolving health needs. A key finding of the review relates to the importance of continuous learning, flexibility and a reflective mindset to align practice with societal needs. However, this is achievable only if health sciences education standards set by accreditation bodies and developed in curricula by tertiary institutions, explicitly incorporate social accountability as a core concern rather than an optional addition. Curriculum developers should collaborate with professional bodies to determine specific core social accountability competencies required across healthcare professions and clinical settings. Consistent with professional development, professional identity formation (DeMatteo & Reeves 2013) should include technical skills plus a commitment to ethical practice, community needs and the ability to work collaboratively across diverse teams (Matthews, Bialocerkowski & Molineux 2019). Healthcare professionals should advocate for their profession and improve the quality of services and patient care by understanding the roles of other HCPs, making referrals and fostering interpersonal collaboration (Du Toit et al. 2019).

Interdisciplinary teamwork is a cornerstone for improving healthcare outcomes and promotes holistic care by leveraging diverse expertise (Bates et al. 2023). Interdisciplinary teamwork also aligns with the goals of global health initiatives that emphasise ‘health for all’ through shared responsibility and collective action (Liu et al. 2015). Interprofessional and stakeholder collaboration cultivates mutual respect, interdisciplinary problem-solving and joint accountability for patient and population outcomes (Clithero et al. 2017). Professional development related to social accountability prepares health sciences graduates for competent and effective service delivery and leadership in policy, equity, advocacy and justice.

### Equity, advocacy and social determinants of health

The inclusion of equity-focused content related to the social determinants of health (SDH), human rights and sustainability (Armstrong & Rispel 2015; Van Wyk et al. 2016; Velardo 2018) addresses primary drivers that produce health disparities rather than focusing on symptoms at an individual level. Curricula can prepare HCPs to be agents of change (Sekome et al. 2023) who advocate for marginalised populations and challenge health systems that perpetuate disparities and inequities by cultivating an understanding of systemic inequities such as those related to race, socio-economic status and culture (Sharma & Kuper 2017). Reflexivity, anti-racism and cultural competence are vital to navigate complex social dynamics and advocate effectively for marginalised groups (Ramsay et al. 2020). Health science education and training that models advocacy, community responsiveness, and engages with priority health concerns of the population is essential (Liu et al. 2015). The shift supports quality service delivery and ethical clinical practice alongside improved population health outcomes. Global health initiatives further recognise the importance of reorienting health services towards prevention and primary healthcare through the growing recognition of interconnected health challenges. However, awareness alone is insufficient. The incorporation of the health inequities and advocacy into curricula requires transformative pedagogies that facilitate critical self-reflection and practical advocacy skills (Chiutsi, Suleman & Perumal-Pillay 2022). Curriculum developers should ensure that theoretical content is supported by experiential learning, which meaningfully engages students with complex social realities.

### Relevance: Community engagement and experiential learning

Community engagement and experiential learning consistently emerge as critical approaches that emphasise the active partnerships of community members in health initiatives. Active community participation not only empowers communities but also promotes health and well-being, directly aligning with the five action areas of health promotion as outlined in the 1986 Ottawa charter (WHO 1986). Curricula should encourage community engagement and active community participation through experiential learning to bridge the gap between theory and practice. Wang (2024) emphasises that curricula should encompass not only theoretical frameworks but also their practical applications within real-world contexts. Similarly, Schwab (2013) highlights that curricula should address tangible, context-specific challenges, encountered in teaching and learning. The practical engagement immerses students in the cultural context of the communities that they serve, which requires an understanding of how race, culture, health systems, policies and global influencing factors such as refugee health, climate change, wars and political discourse shape health outcomes (Kelly et al. 2022).

Experiential learning, particularly when conducted in partnership with empowered communities, creates reflective spaces where students recognise how context-specific challenges, such as subtle manifestations of racism, shape access to care, patient experience and health outcomes. Practically and openly engaging with communities moves beyond abstract concepts of racial awareness and cultural sensitivity towards fostering deep race consciousness and an explicit understanding of how racism operates institutionally and interpersonally to produce health inequities. Race consciousness goes beyond mere cultural differences towards an explicit understanding of how race and racism impact the experience of communities and their health outcomes (Sharma & Kuper 2017). An important aspect of experiential learning involves empowering students to address these tensions without feeling threatened. In practice, this means curricula should not only teach cultural competence but also develop students’ abilities to identify, analyse and challenge racial inequities within healthcare systems.

A key recommendation for curriculum developers includes designing experiential learning modules that include direct engagement with marginalised groups such as communities of colour, people with disabilities, refugees and underprivileged populations. The modules should integrate guided reflection on the history and ongoing impact of social determinants of health, such as racism in healthcare, supported by active community participation in all initiatives. Such approaches, exemplified by rural immersion and service learning projects (Emadzadeh et al. 2016), centre the voices and lived experiences of those affected by health disparities, equipping students with practical skills to confront inequities and contribute to inclusive health practices.

### Equity: Social justice and transformative education

Social justice emerges as a unifying element and is grounded in the belief that every individual and group within a given society has a right to civil liberties, equal opportunities, fairness, and participation in the educational, economic, social and moral freedoms and responsibilities valued by the community. Social justice plays a crucial role in addressing health inequities, guiding how interventions are identified and implemented towards health issues within populations. The element of social justice shapes curricula by guiding what is taught as well as preparing HCPs to ethically respond to systemic barriers resulting in health disparities.

The review highlights that curricula grounded in social justice focus on transformative education strategies, such as experiential learning and critical self-reflection, which are essential to developing HCPs who challenge health disparities and system-level barriers (Mlambo et al. 2018). Social justice strategies are pivotal in shaping HCPs who are empathetic, socially conscious and adaptable to evolving healthcare challenges. Social justice in the curricula also encompasses the development of skills like compassion, leadership, resilience and a commitment to the advancement and protection of human rights within clinical and community settings.

The integration of social justice in curricula will develop HCPs who not only deliver competent and effective healthcare but who actively work to reshape health systems. Embedding social justice in undergraduate health sciences curricula equips HCPs to be accountable to the communities that they serve, aligning with the global agenda for health equity and sustainable healthcare systems.

### Implementation strategies and barriers

Social accountability can be operationalised by integrating courses in anthropology, epidemiology and public health with a focus on poverty and health disparities (Abdalla et al. 2022). Partnerships must be formed between health science schools and key stakeholders such as policy-makers and community representatives to promote community-oriented strategies and experiential learning (Shrivastava, Shrivastava & Wanjari 2024). Education institution accreditation standards should align with social accountability goals, ensuring that graduates are competent in population health and working with marginalised populations.

However, barriers exist to the inclusion of social accountability in the curriculum as well as the practical implementation of the elements. Barriers relate to limited curricula exposure, a lack of time to teach all of the identified elements, and the lack of staff expertise and enthusiasm for elements of social accountability (Benrimoh et al. 2016). Practical implementation of social accountability may be hindered by overworked and overwhelmed HCPs, resource constraints (Anawati et al. 2023), inadequate capacity building, a shortfall in community participation, safety concerns within the communities and a lack of community-oriented programmes (Clithero-Eridon et al. 2020).

Furthermore, a critical concern in implementing social accountability within curricula relates to who delivers the content. Evidence has demonstrated that the method of instruction (Schwab 2013; Schwab et al. 2022; Wang 2024) as well as the educators themselves (Mlambo et al. 2018; Sharma & Kuper 2017) influences how social accountability is internalised by students.

### Implications for practice

The results from the scoping review lay the foundation for curriculum re-design by emphasising that undergraduate health sciences curricula must incorporate experiential, service-learning opportunities together with theoretical teaching that emphasise primary healthcare principles, SDH, and equity-focused interventions. In addition, educators and those training health sciences students should be from diverse backgrounds and have knowledge around both health and social sciences, which can enrich learning experiences. It also highlighted that continuous development in areas such as communication skills, diversity, cultural humility, inclusivity, advocacy and interdisciplinary teamwork is essential for fostering lifelong learning among HCPs. Lastly, HCPs should be empowered to understand policies within their health system and work towards advocating for healthy public policies.

### Limitations of current literature

The scoping review provides valuable insights, but several gaps remain, such as limited focus on evaluating the long-term impact of student-led community-based interventions on patient outcomes, insufficient exploration of structural barriers to implementing equity-focused curricula in diverse geographic contexts and a lack of standardised frameworks for assessing competencies related to SDH and interdisciplinary collaboration. Furthermore, most of the studies focused on medical students or nursing students, with only a few relating to rehabilitation professionals.

## Conclusion

The scoping review highlights the multifaceted nature of social accountability in undergraduate health sciences curricula, encompassing the core values of relevance, cost-effectiveness, quality and equity. Key elements such as community engagement, culturally and clinically competent service delivery, advocacy for social justice and efficient use of resources are critical to prepare HCPs to meet the needs of the communities that they serve. The review underscores the need for equity-driven approaches in healthcare systems and tertiary institutions, emphasising interprofessional collaboration, transformative and decolonial learning, leadership development and self-reflection to foster socially accountable HCPs. Additionally, including community-oriented and global health literacies within curricula improves alignment with national and international health priorities. Emphasising experiential learning through active community involvement bridges the gap between theory and practice, while addressing the broader socio-political and environmental determinants of health. Together, the elements provide a comprehensive framework to develop HCPs who deliver equitable, relevant, cost-effective and quality services that are responsive to the evolving health needs of populations served.
